# Publisher Correction: ATP-citrate lyase promotes axonal transport across species

**DOI:** 10.1038/s41467-021-26998-y

**Published:** 2021-11-12

**Authors:** Aviel Even, Giovanni Morelli, Silvia Turchetto, Michal Shilian, Romain Le Bail, Sophie Laguesse, Nathalie Krusy, Ariel Brisker, Alexander Brandis, Shani Inbar, Alain Chariot, Frédéric Saudou, Paula Dietrich, Ioannis Dragatsis, Bert Brone, Loïc Broix, Jean-Michel Rigo, Miguel Weil, Laurent Nguyen

**Affiliations:** 1 Laboratory for Neurodegenerative Diseases and Personalized Medicine, The Shmunis School of Biomedicine and Cancer Research, The George S. Wise Faculty for Life Sciences, Sagol School of Neurosciences, Tel Aviv University, Ramat Aviv, 69978 Israel; 2 Laboratory of Molecular Regulation of Neurogenesis, GIGA-Stem Cells, Interdisciplinary Cluster for Applied Genoproteomics (GIGA-R), University of Liège, C.H.U. Sart Tilman, Liège, 4000 Belgium; 3 BIOMED Research Institute, Hasselt, 3500 Belgium; 4 Life Sciences Core Facilities, Weizmann Institute of Science, Rehovot, Israel; 5 Laboratory of Medical Chemistry, GIGA-Stem Cells, Interdisciplinary Cluster for Applied Genoproteomics (GIGA-R), University of Liège, C.H.U. Sart Tilman, Liège, 4000 Belgium; 6 Univ. Grenoble Alpes, Inserm, U1216, CHU Grenoble Alpes, Grenoble Institut Neurosciences, 38000 Grenoble, France; 7 Inserm, U1216, F-38000 Grenoble, France; 8 CHU Grenoble Alpes, F-38000 Grenoble, France; 9 Department of Physiology, University of Tennessee Health Science Center, Memphis, TN 38163 USA

**Keywords:** Protein transport, Molecular neuroscience

Correction to: *Nature Communications* 10.1038/s41467-021-25786-y, published online 7 October 2021.

This Article contained an error in the author list. The original author list omitted Silvia Turchetto as an equally contributing first author. This has been corrected in the author list and author contribution statement. The PDF and HTML versions of the Article have been updated.

