# Supplementary material for: ATP-citrate lyase promotes axonal transport across species
Source: Nat Commun. 2021 Oct 7;12:5878. doi: 10.1038/s41467-021-25786-y (PMC8497606; doi:10.1038/s41467-021-25786-y)
Supplement: Supplementary file 1 — Supplementary Information [file 41467_2021_25786_MOESM1_ESM.pdf]

Supplementary Figures for Even et al.

Supplementary Figure 1

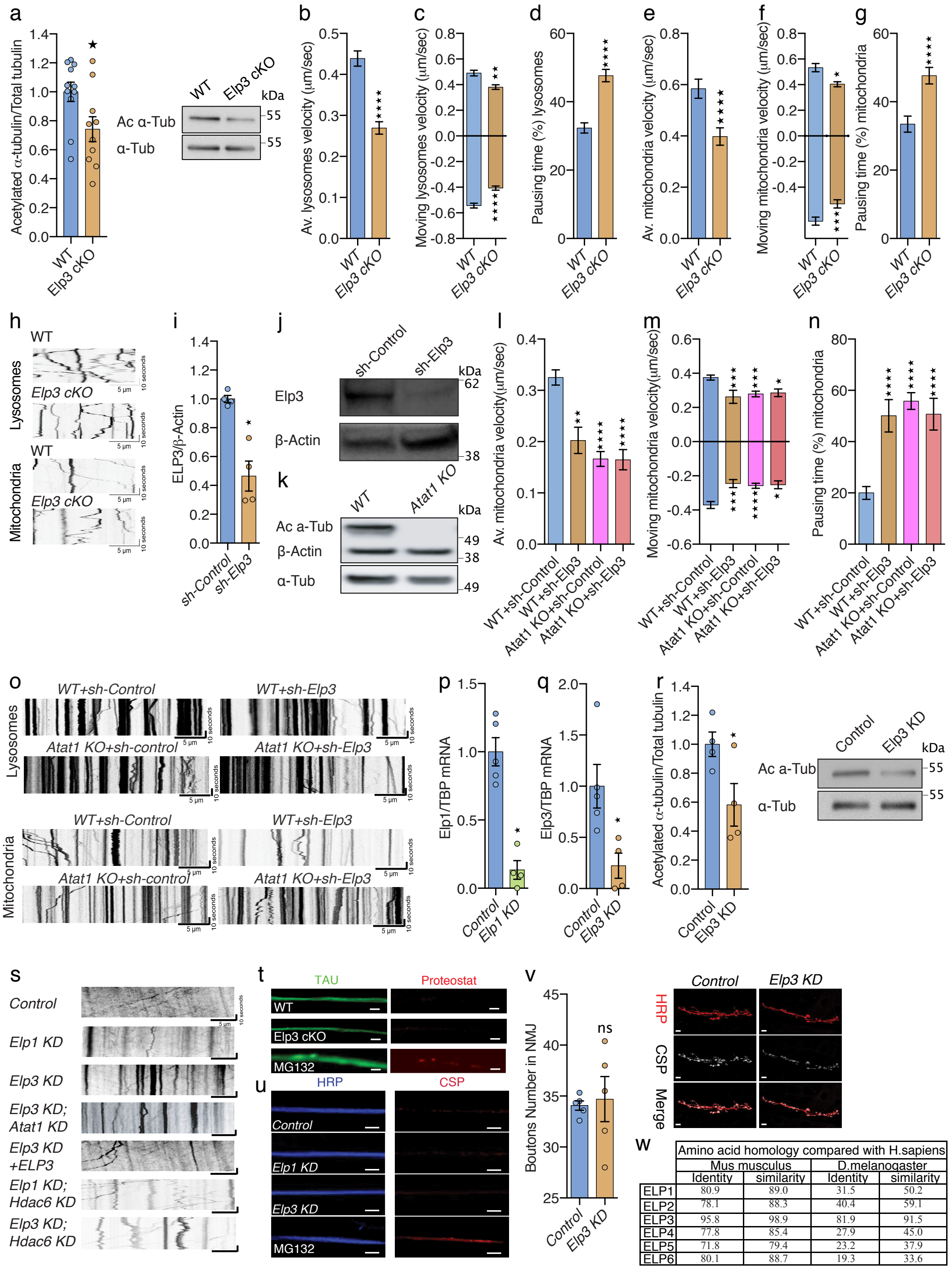

	Amino acid homology compared with H.sapiens			
	Mus musculus		D.melanogaster	
	Identity	similarity	Identity	similarity
ELP1	80.9	89.0	31.5	50.2
ELP2	78.1	88.3	40.4	59.1
ELP3	95.8	98.9	81.9	91.5
ELP4	77.8	85.4	27.9	45.0
ELP5	71.8	79.4	23.2	37.9
ELP6	80.1	88.7	19.3	33.6

Supplementary Fig. 1: Kymographs of motile lysosomes, mitochondria and Synaptotagmin-GFP. RNAi targeting efficiency and neuromuscular junction assessment in fly model. (**a**) Immunoblotting and histogram of acetylated α-tubulin (Ac α-Tub), total α tubulin (Tot α-Tub)

in newborn *WT or Elp3cKO* mice cortical brain extracts. (**b-h**) Axonal transport recordings of PNs isolated from E14.5 WT or *Elp3 cKO* embryos and cultured for 5 DIV in microfluidic devices to analyze average (av.) velocity (**b, e**), moving velocity (**c, f**) and percentage of pausing time (**d, g**) of lysosomes (LysoTracker®) and mitochondria (MitoTracker®). (**h**) Representative kymographs of moving lysosomes (**top**) and mitochondria (**bottom**) in E14.5 in *WT or Elp3cKO* mice PNs axons cultured for 5 DIV in microfluidic devices. Scale bars are 10 seconds and 5 µm. (**i-j**) Immunoblotting and quantification of Elp3 and ß-Actin in cortical neurons transfected with sh-*Elp3* or sh-*Control*. (**k**) Immunoblotting of acetylated α-tubulin (Ac α-Tub), total α-tubulin (Tot α-Tub) and ß-Actin in extracts from P0 WT or *Atat1* KO brains. (**l-n**) Axonal transport analysis of mitochondria (MitoTracker®) in WT or *Atat1* KO neurons infected either with sh-Control or sh-*Elp3* to analyze average (av.) velocity (**l**), moving velocity (**m**) and percentage of pausing time (**n**). (**o**) Representative kymographs of lysosomes (**top**) and mitochondria (**bottom**) in E14 in WT or *Atat1 KO* neurons infected either with sh-Control or sh-*Elp3* mice cortical neurons axons cultured for 5 DIV in microfluidic devices. Scale bars are 10 seconds and 5 µm. (**p-q**) mRNA levels of *Elp1* and *Elp3* from fly head extracts, expressing RNAi under the pan-neuronal driver (Elav:GAL4) in control, *Elp1* KD (**p**) and *Elp3* KD (**q**). (**r**) Immunoblotting and histogram of acetylated α-tubulin (Ac α-Tub), total α-tubulin (Tot α-Tub) from fly head extracts, expressing RNAi under the pan-neuronal driver (Elav:GAL4) in control and *Elp3* KD. (**s**) Representative kymographs of Synaptotagmin-GFP (Syt1-GFP) in motoneurons from 3rd instar larvae of *Drosophila melanogaster* expressing RNAi under a motoneuron-specific driver (D42:GAL4); control, *Elp1* KD, *Elp3* KD, *Atat1; Elp3* KD, *Elp3* KD + human *ELP3 (ELP3), Elp1;Hdac6* KD and *Elp3;Hdac6* KD. Scale bars are 10 seconds and 5 µm. (**t**) Protein aggregation was estimated in axons (Tau+; in green) of cultured mice cortical neurons of WT, *Elp3cKO* and MG132 treated neurons (positive control) by the chemical dye Proteostat© as marker for protein aggregation (in red). Scale bar is 10 µm.

(**u**) Protein aggregation was estimated in motor neurons exiting the ventral ganglion of 3rd instar larvae expressing RNAi under motoneurons specific driver (D42:GAL4); control, *Elp1* KD, *Elp3* KD and *MG132 treatment* (positive control) by immunolabeling of cysteine string protein (CSP+; red) as marker for aggregation, and the neuronal marker horseradish peroxidase (HRP+; blue). Scale bar is 10 µm. (**v**) Representative images (**right**) of neuromuscular junctions in 3rd instar larvae immunolabeled for cysteine string protein (CSP) and horseradish peroxidase (HRP) and a histogram of its quantification (**left**). (**w**) Table comparing the amino acid homology of the mouse and fly Elongator complex subunits (ELP1-6) to the human sequence, by sequence identity and similarity. Description of graphical summaries here within are histograms of means ± SEM. Significance was determined by: (**a, r**) two-sided t-test, (**b, c, d, e, f, g, i, p, q, v**) two-sided Mann-Whitney test, and (**l, m, n**) two-sided Kruskal Wallis one-way ANOVA. Specifically, [(**a**) p = 0.0270, t = 2.396, df = 19; (**b**) p < 0.0001, U = 111906; (**c**) p < 0.0001, U = 71910 and p = 0.0029, U = 45529 for anterograde and retrograde, respectively; (**d**) p < 0.0001, U = 68287; (**e**) p < 0.0001, U = 21672; (**f**) p = 0.0349, U = 20092 and p = 0.0002, U = 27975 for anterograde and retrograde, respectively; (**g**) p < 0.0001, U = 21907; (**i**) p = 0.0286, U = 0 ; (**l**) p < 0.0001, K = 67.88; (**m**) p < 0.0001, K = 25.42 and p < 0.0001, K = 25.89 for anterograde and retrograde, respectively; (**n**) p < 0.0001, K = 73.34; (**p**) p = 0.0159, U = 0; (**q**) p = 0.0159, U = 0; (**r**) p = 0.0487, t = 2.467, df = 6**;** (**v**) p = 0.6905, U = 10]. In addition, the post hoc multiple comparisons, to analyze statistical difference of each condition compared to control for (**l, m, n**) are Dunn's test, and are *p < 0.05, **p < 0.01, ***p < 0.001, and ****p < 0.0001. (a) Number of mice: WT n = 11; Elp3 cKO n = 10. (**b**) Number of vesicles: WT n = 656; Elp3 cKO n = 423. 5 mice per group. (**c**) Number of vesicles: WT n = 417 (anterograde), n = 566 (retrograde); Elp3 cKO n = 254 (anterograde), n = 327 (retrograde). 5 mice per group. (**d**) Number of vesicles: WT n = 506; Elp3 cKO n = 366. 5 mice per group. (**e**) Number of vesicles: WT n = 239; Elp3 cKO n = 232. 5 mice per group. (**f**)

Number of vesicles: WT n = 209 (anterograde), n = 240 (retrograde); Elp3 cKO n = 218 (anterograde), n = 287 (retrograde). 5 mice per group. (**g**) Number of vesicles: WT n = 239; Elp3 cKO n = 232. 5 mice per group. (**i**) 4 animals per group. (**l**) Number of vesicles: WT+sh-Control n = 201; WT+sh-Elp3 n = 43; Atat1 KO+sh-Control n = 149; Atat1 KO+sh-Elp3 n = 42. 3 mice per group. (**m**) Number of vesicles: WT+sh-Control n = 112 (anterograde), n = 90 (retrograde); WT+sh-Elp3 n = 22 (anterograde), n = 34 (retrograde); Atat1 KO+sh-Control n = 64 (anterograde), n = 71 (retrograde); Atat1 KO+sh-Elp3 n = 30 (anterograde), n = 18 (retrograde). 3 mice per group. (**n**) Number of vesicles: WT+sh-Control n = 201; WT+sh-Elp3 n = 46; Atat1 KO+sh-Control n = 149; Atat1 KO+sh-Elp3 n = 42. 3 mice per group. (**p**) Number of fly brains: Control n = 5; Elp1 KD n = 4; (**q**) Number of fly brains: Control n = 5; Elp3 KD n = 4. **(r)** Number of fly brains: Control n = 4; Elp3 KD n = 4; (**v**) Number of 3rd instar larvae: Control n = 5; lp3 KD n = 5. Source data are provided with this paper.

Supplementary Figure 2

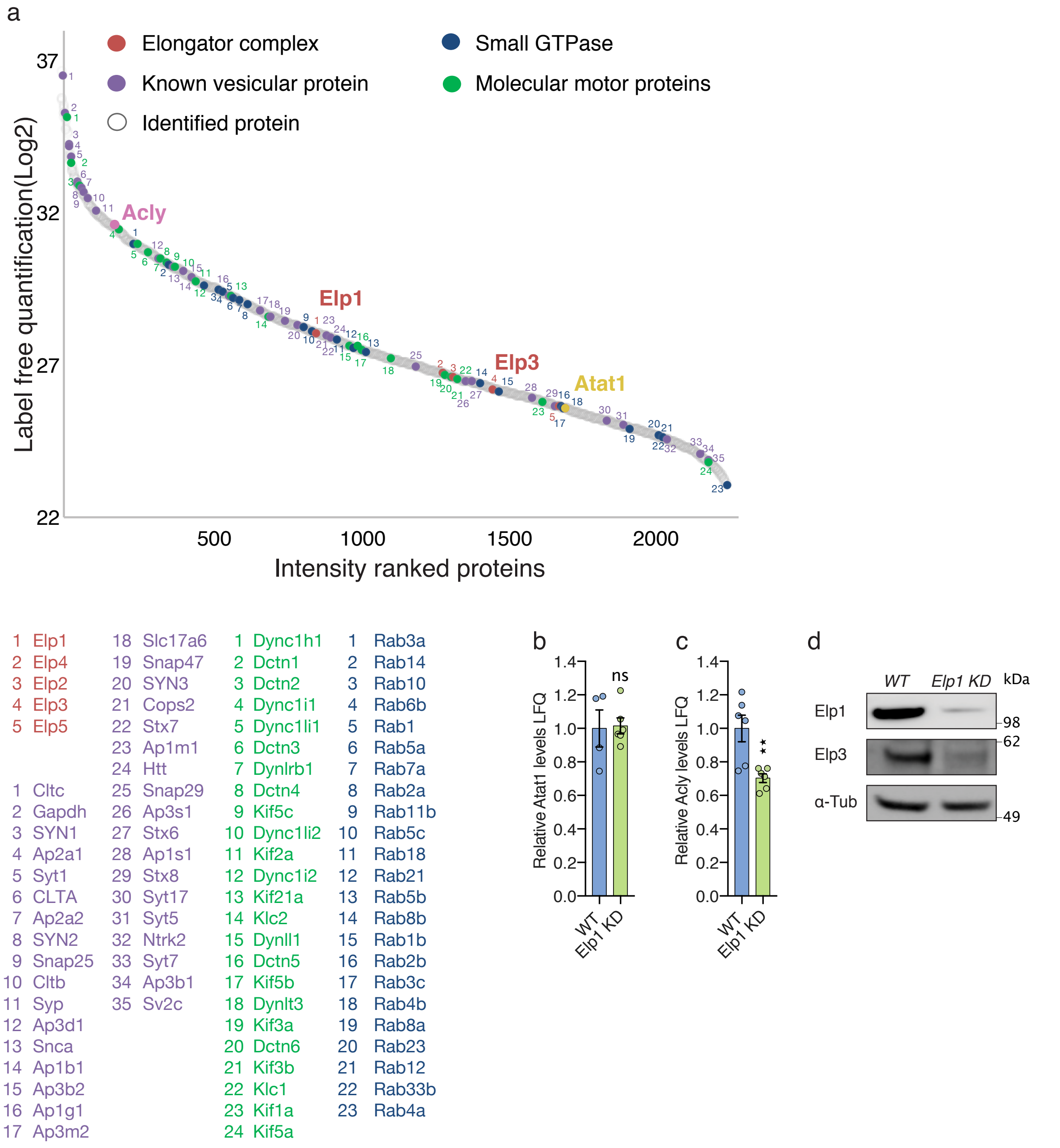

Supplementary Fig. 2: LC-MS/MS and Acly levels in E14.5 cortical brains. (a) LC-MS/MS proteomic analysis of vesicular fraction isolated from adult brain cortices of WT and *Elp1 KD* mice, proteins were ranked by intensity and plotted according to their relative abundance (gray spots). Acly (pink), Atat1 (yellow), and Elongator subunits (red) detection among proteins previously identified as vesicular components (purple), small GTpase (blue) and molecular

motors (green) (n = 3, graph represent the mean intensity value). (**b-c**) Histograms of relative Atat1 and Acly label-free quantification (LFQ) intensities analyzed by LC-MS/MS of P3 fraction from WT and *Elp1 KD* mice. (**d**) Immunoblotting to detect Elp1, Elp3 and α-tubulin (α-Tub) in cortical extracts from adult WT and *Elp1 KD* mice. Description of graphical summaries here within are histograms of means ± SEM. Significance was determined by two-sided Mann-Whitney test (**b,c**). Specifically, [(**g**) p = 0.7619, U = 10; (**h**) p = 0.0087]. Number of mice: WT n = 4; Elp1 n = 6 (**b**); WT n = 6; Elp1 KD n = 6 (**c**). Source data are provided with this paper.

Supplementary Figure 3

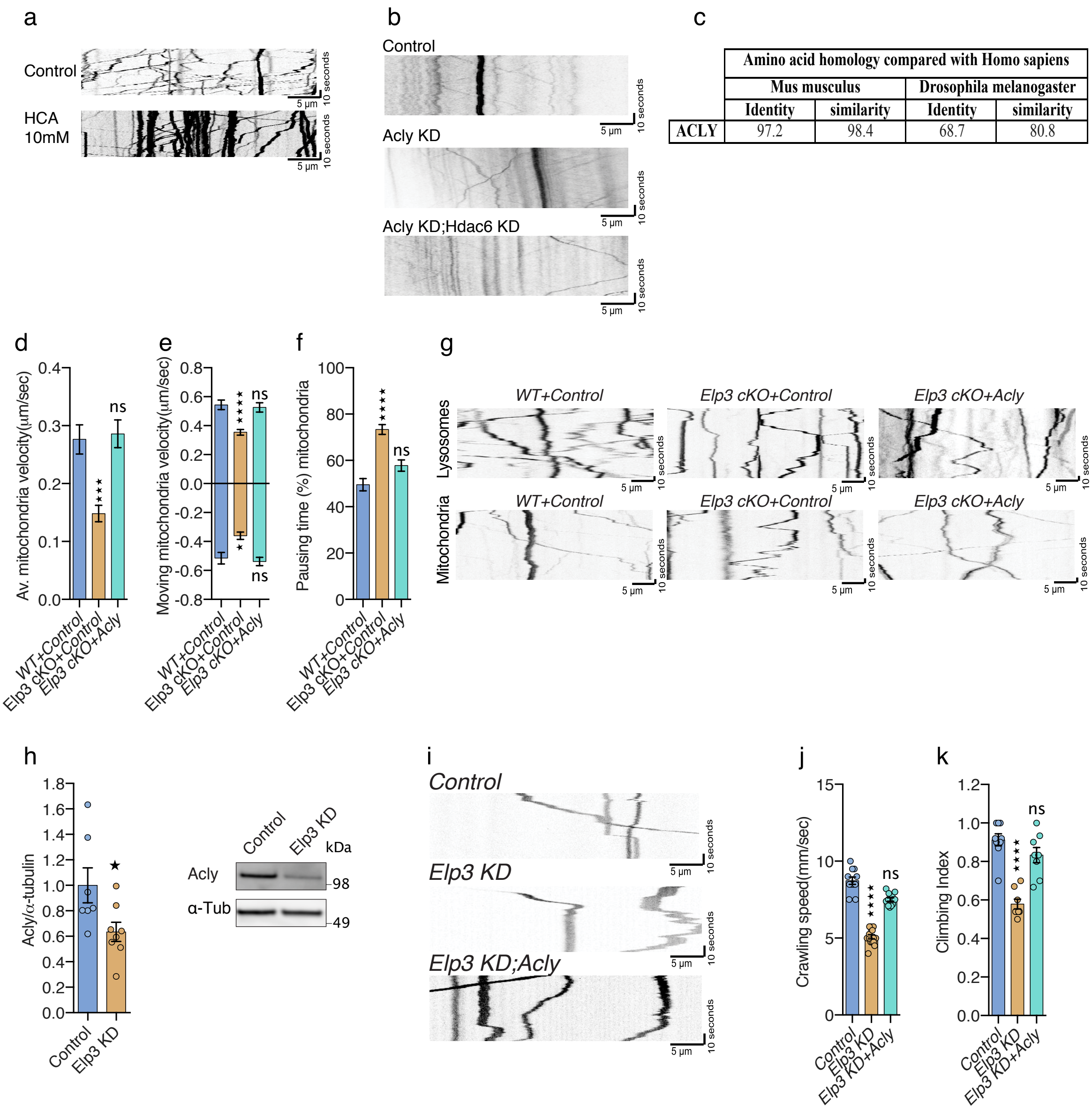

Amino acid homology compared with Homo sapiens				
	Mus musculus		Drosophila melanogaster	
	Identity	similarity	Identity	similarity
ACLY	97.2	98.4	68.7	80.8

Supplementary Fig. 3: Expression of Acly/ACLY in mice cultured neurons and fly heads. Kymographs after time-lapse recording of motile lysosomes, mitochondria and Synaptotagmin-GFP+ vesicles (**a**,**b**). (**a**) Representative kymographs of lysosomes in E14.5 mice cortical neuron axons cultured for 5 DIV in microfluidic devices and incubated with 10mM hydroxycitrate acid (HCA) for 8 hours. Scale bars are 10 seconds and 5 µm. (**b**) Representative kymographs of Synaptotagmin-GFP (Syt1-GFP) in motoneurons from 3rd instar larvae of *Drosophila melanogaster* expressing RNAi under a motoneuron-specific driver

(D42:GAL4); control, *Acly KD* and *Acly;Hdac6 KD*. Scale bars are 10 seconds and 5 µm. (**c**) Table comparing the amino acid homology of the mouse and fly Acly to the human, by sequence identity and similarity. (**d-f**) Time-lapse recordings of axonal transport of mitochondria (MitoTracker®) from E14.5 cortical neurons of *WT or Elp3cKO* mice cultured 5 DIV in microfluidic devices transfected with control or *Acly* expressing constructs, to analyze average (av.) velocity (**d**), moving velocity (**e**) and percentage of pausing time (**f**). (**g**) Representative kymographs of lysosomes (**top**) and mitochondria (**bottom**) in E14.5 *WT or Elp3 cKO* mice cortical neuron axons transfected with Control or ACLY expressing construct and cultured 5 DIV in microfluidic devices. Scale bars are 10 seconds and 5 µm. (**h**) Immunoblotting and quantification of Acly and total tubulin expressions in *Drosophila melanogaster* head extracts from control or Elp3 RNAi (Elp3 KD) (under the pan-neuronal driver, Elav:GAL4) adult flies. (**i**) Representative kymographs of Synaptotagmin-GFP (Syt1-GFP) in 3rd instar larvae motor neurons expressing RNAi under a motoneuron-specific driver (D42:GAL4); control, *Elp3* KD and *Elp3* KD + *Acly*. Scale bars are 10 seconds and 5 µm. (**j-k**) Locomotion assays; histograms of climbing index of adult flies (**j**) and crawling speed of 3rd instar larvae (**k**), genotypes as indicated on graphs. Description of graphical summaries here within are histograms of means ± SEM. Significance was determined by: (**d, e, f**) two-sided Kruskal Wallis one-way ANOVA, (**h**) two-sided t-test, and (**j, k**) two-sided one-way analysis of variance (ANOVA). Specifically, [(**d**) $p < 0.0001$, K = 27.47; (**e**) $p < 0.0001$, K = 26.16 and $p = 0.0003$, K = 16.04 for anterograde and retrograde, respectively; (**f**) $p < 0.0001$, K = 49.32; (**h**) $p = 0.031$, $t = 2.407$, df = 13; (**j**) $p < 0.0001$, $F = 28.45$; (**k**) $p < 0.0001$, $F = 139.5$]. In addition, the post hoc multiple comparisons, to analyze statistical difference of each condition compared to control for (**d, e, f, j,k**) are Dunn's test, (**j,k**) Dunnet's, and are *$p < 0.05$, ***$p < 0.001$, and ****$p < 0.0001$. (**d**) Number of vesicles: WT+Control n = 318; Elp3 cKO+Control n = 383; Elp3 cKO+Acly n = 240. 5 mice per group. (**e**) Number of vesicles: WT+Control n =

87 (anterograde), n = 67 (retrograde); Elp3 cKO+Control n = 94 (anterograde), n = 54 (retrograde); Elp3 cKO+Acly n = 51 (anterograde), n = 63 (retrograde). 5 mice per group. (**f**) Number of vesicles: WT+Control n = 317; Elp3 cKO+Control n = 383; Elp3 cKO+Acly n = 379. 5 mice per group. (**h**) Number of larvae: Control n = 7; Elp3 KD n = 8. (**j**) Number of larvae: Control n = 11; Elp3 KD n = 15; Elp3 KD+Acly n = 12. (**k**) Number of larvae: Control n = 9; Elp3 KD n = 8; Elp3 KD+Acly n = 8. Source data are provided with this paper.

Supplementary Figure 4

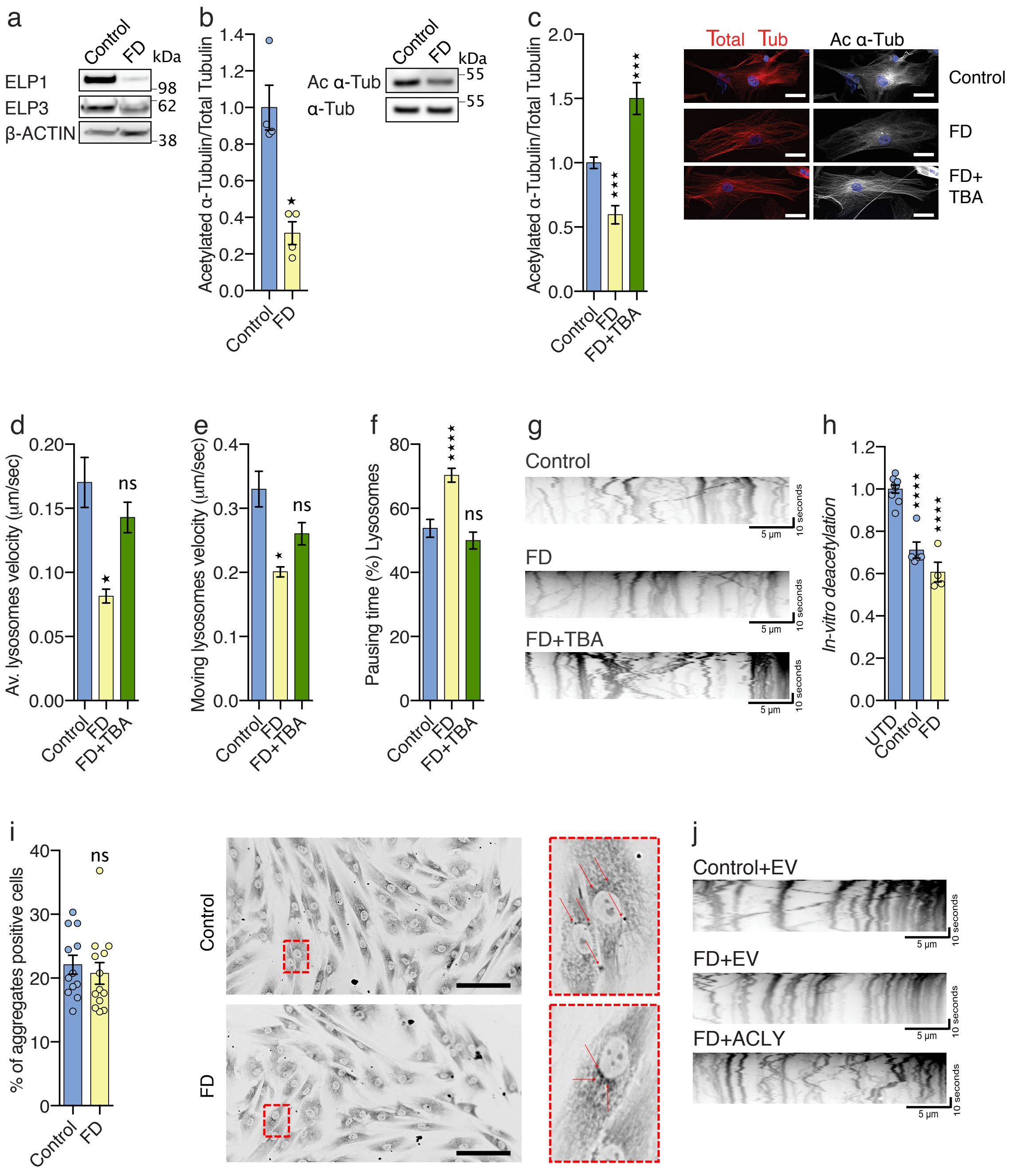

Supplementary Fig. 4: Pharmacological and genetic rescue of vesicular transport in FD fibroblasts. (a) Immunoblotting of ELP1, ELP3 and ß-ACTIN in human primary fibroblasts from healthy control and FD patients. (**b**) Immunoblotting and quantification of acetylated α-tubulin (Ac α-Tub), total α-tubulin (Tot α-Tub) in extracts from cultured controls or FD human primary fibroblasts. (**c**) Immunolabelings and quantification of acetylated α-tubulin (Ac α-Tub)

and total tubulin (Tot α-Tub) in human primary fibroblasts from controls and FD patients incubated with vehicle or Tubastatin (TBA). Scale bar is 50 µm. (**d-f**) Recording of moving lysosomes using live fluorescent probe (LysoTracker®) in primary control or FD cultured fibroblasts incubated with vehicle (DMEM media) or TBA to analyze average (av.) velocity (**d**), moving velocity (**e**) and percentage of pausing time (**f**). (**g**) Representative kymographs of transported lysosomes in human primary fibroblasts from healthy controls or FD patients incubated with vehicle (DMEM media) or Tubastatin (TBA). Scale bars are 10 seconds and 5µm. (**h**) In vitro deacetylation assay of endogenously acetylated bovine brain tubulin incubated for 4 hours with extracts of control and FD cultured fibroblasts or without tissue extract (control). (**i**) Protein aggregation was estimated in fibroblasts from healthy controls and FD patients after incubation with the chemical dye Proteostat©, a marker for protein aggregation (in black, see also red arrows). Scale bar is 50 µm. (**j**) Representative kymographs of transported lysosomes in human primary fibroblasts from healthy controls or FD patients transfected with ACLY or Control expressing constructs. Scale bars are 10 seconds and 5µm. Description of graphical summaries here within are histograms of means ± SEM. Significance was determined by: (**b, i**) two-sided Mann-Whitney test, (**c**) two-sided one-way analysis of variance (ANOVA), (**d, e, f**) two-sided Kruskal Wallis one-way ANOVA, and (**h**) two-sided one-way ANOVA. Specifically, [(**b**) $p = 0.0286$, $U = 0$ (**c**) $p < 0.0001$, $F = 30.36$; (**d**) $p < 0.0001$, $K = 24.26$; (**e**) $p < 0.0001$, $K = 23.82$; (**f**) $p < 0.0001$, $K = 44.79$; (**h**) $p < 0.0001$, $F = 48.27$ (**i**) $p = 0.3398$, $U = 60$]. In addition, the post hoc multiple comparisons, to analyze statistical difference of each condition compared to control for (**c**) is Dunnett's test, for (**d, e, f**) are Dunn's test, and are *$p < 0.05$, ***$p < 0.001$, and **** $p < 0.0001$. (**a**) Lysates from 5 human primary fibroblast lines from control and FD patients. (**b**) Lysates from 4 human primary fibroblast lines from control and FD patient. (**c**) Number of cells: Control n = 18; FD n = 30; FD+TBA n = 13. 5 human primary fibroblast lines from control and FD patients. (**d**)

Number of vesicles: Control n = 229; FD n = 273; FD+TBA n = 257. 5 human primary fibroblast lines from control and FD patients. (**e**) Number of vesicles: Control n = 174; FD n = 190; FD+TBA n = 202. (**f**) Number of vesicles: Control n = 237; FD n = 271; FD+TBA n = 267. (**h**) Lysates from human primary fibroblasts from control (n = 5) and FD (n = 4) patients. (**i**) Number of cells: Control n = 12; FD n = 13. Source data are provided with this paper.

Supplementary Figure 5

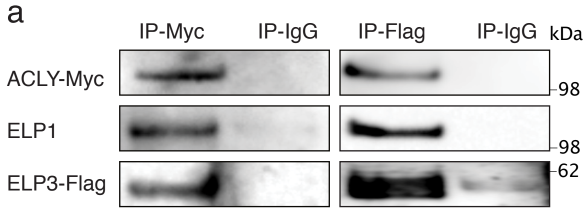

Supplementary Fig. 5: In vitro interaction between the Elongator subunits ELP1 and ELP3 with ACLY. (a) Western blotting with Flag or Myc antibodies showing co-immunoprecipitation between endogenous ELP1 or ELP3-Flag and ACLY-Myc in extracts from HEK293 cells co-transfected with ACLY-Myc and ELP3-Flag. IgG antibodies were used as Control. Source data are provided with this paper.

Supplementary Table 1
List of Antibodies used for western blotting

Protein	Company	Cat #	WB	IF	ELISA	IP
α-Tubulin	Sigma-Aldrich	T9026	1:5,000		1:2,000	
α/β-Tubulin (total tubulin)	Cytoskeleton	ATN02-A		1:150		
Tubulin YL1/2	Abcam	ab6160		1:1,000		
Acetylated α-Tubulin	Sigma-Aldrich	T7451	1:15,000	1:15,000		
β-Tubulin	Cell Signaling	2146		1:100		
β-actin	Sigma-Aldrich	A3853	1:20,000			
GAPDH	Millipore	MAB374	1:1,000			
HDAC6	Santa Cruz Biotechnology	sc-5258	1:200			
Atat1	Max Nachury		1:1000			
Tau-1	Millipore	MAB3420		1:500		
CSP	DSHB	DCSP-2 (D6D)		1:10		
Elp1/IKAP	Anaspec	AS-54494	1:500			
Elp3	Jesper Svejstrup		1:1,000			
HH3	Cell Signaling	9715	1:1,000			
Synaptophysin	Sigma-Aldrich	s5768	1:1,000			
Synaptophysin-1	Synaptic Systems	101004	1:1,000			

Acly	Aviva Systems Biology	OAGA04026		1:100		
Acly	Cell signaling	13390	1:1,000			
AceCS1	Cell Signaling	3658	1:1,000	1:100		
Myc	Cell Signaling	#2276				1:1,000
FLAG	Sigma-Aldrich	F3165				1:600
Goat anti mouse	Jackson ImmunoResearch Labs	115-035-003	1:10,000	1:200	1:5,000	
Goat anti rabbit	Jackson ImmunoResearch Labs	111-035-003	1:10,000	1:200		
Donkey anti goat	Jackson ImmunoResearch Labs	705-035-003	1:10,000			
