# Supplementary material for: ATP-citrate lyase promotes axonal transport across species
Source: Nat Commun. 2021 Oct 7;12:5878. doi: 10.1038/s41467-021-25786-y (PMC8497606; doi:10.1038/s41467-021-25786-y)
Supplement: Supplementary file 3 — Reporting Summary [file 41467_2021_25786_MOESM3_ESM.pdf]

nature research

Corresponding author(s): Laurent Nguyen
Last updated by author(s): 13/08/2021

Reporting Summary

Nature Research wishes to improve the reproducibility of the work that we publish. This form provides structure for consistency and transparency in reporting. For further information on Nature Research policies, see our Editorial Policies and the Editorial Policy Checklist.

Statistics

For all statistical analyses, confirm that the following items are present in the figure legend, table legend, main text, or Methods section.

n/a	Confirmed	
☐	☒	The exact sample size (n) for each experimental group/condition, given as a discrete number and unit of measurement
☐	☒	A statement on whether measurements were taken from distinct samples or whether the same sample was measured repeatedly
☐	☒	The statistical test(s) used AND whether they are one- or two-sided<br>*Only common tests should be described solely by name; describe more complex techniques in the Methods section.*
☐	☒	A description of all covariates tested
☐	☒	A description of any assumptions or corrections, such as tests of normality and adjustment for multiple comparisons
☐	☒	A full description of the statistical parameters including central tendency (e.g. means) or other basic estimates (e.g. regression coefficient) AND variation (e.g. standard deviation) or associated estimates of uncertainty (e.g. confidence intervals)
☐	☒	For null hypothesis testing, the test statistic (e.g. F, t, r) with confidence intervals, effect sizes, degrees of freedom and P value noted<br>*Give P values as exact values whenever suitable.*
☒	☐	For Bayesian analysis, information on the choice of priors and Markov chain Monte Carlo settings
☒	☐	For hierarchical and complex designs, identification of the appropriate level for tests and full reporting of outcomes
☒	☐	Estimates of effect sizes (e.g. Cohen's d, Pearson's r), indicating how they were calculated

Our web collection on statistics for biologists contains articles on many of the points above.

Software and code

Policy information about availability of computer code

Data collection	No software was used for data collection.
Data analysis	Fiji plugin-KymoToolBox v1.0.1 (fabrice.cordelieres@curie.u-psud.fr) and StackReg (http://bigwww.epfl.ch/thevenaz/stackreg/).

For manuscripts utilizing custom algorithms or software that are central to the research but not yet described in published literature, software must be made available to editors and reviewers. We strongly encourage code deposition in a community repository (e.g. GitHub). See the Nature Research guidelines for submitting code & software for further information.

Data

Policy information about availability of data

All manuscripts must include a data availability statement. This statement should provide the following information, where applicable:

- Accession codes, unique identifiers, or web links for publicly available datasets
- A list of figures that have associated raw data
- A description of any restrictions on data availability

As detailed in the Data Availability Statement, informations relative to the proteomic data can be accessed at ProteomeXchange with : Project Name: ATP-Citrate lyase fuels axonal transport across species; Project accession: PXD021186; Hyperlink: http://proteomecentral.proteomexchange.org/cgi/GetDataset?ID=PXD021186. The authors declare that all data supporting the findings of this study are available within the paper and its supplementary information files. Raw data and the original pictures of WB membranes are available in the file "Source data".

Field-specific reporting

Please select the one below that is the best fit for your research. If you are not sure, read the appropriate sections before making your selection.

☒ Life sciences ☐ Behavioural & social sciences ☐ Ecological, evolutionary & environmental sciences

For a reference copy of the document with all sections, see nature.com/documents/nr-reporting-summary-flat.pdf

Life sciences study design

All studies must disclose on these points even when the disclosure is negative.

Sample size	Sample size was determined based on previous publications on cortical development and axonal transport (Even at aL,. Sci Adv (2019) Dec 18;5(12):eaax2705; Hinkelmann et al.,. Nat Commun (2016) Oct 24;7:13233).
Data exclusions	No data criteria was performed.
Replication	All results have been replicated independently at least two times and by distinct experimenters.
Randomization	For in vivo and in vitro experiments, time-mated pregnant mice and embryos were randomly allocated to the different treatments during surgeries, experimental procedures and analyses. In vitro experiments with cell lines did not involve sample randomization as they were performed on several biological replicates.
Blinding	Information regarding treatment of each sample was not transferred between the animal surgeon, the microscopist and analyst, until the final data was acquired for analyses. The investigators were blinded during data collection/data analysis.

Reporting for specific materials, systems and methods

We require information from authors about some types of materials, experimental systems and methods used in many studies. Here, indicate whether each material, system or method listed is relevant to your study. If you are not sure if a list item applies to your research, read the appropriate section before selecting a response.

Materials & experimental systems

n/a	Involved in the study	
☐	☒	Antibodies
☐	☒	Eukaryotic cell lines
☒	☐	Palaeontology and archaeology
☐	☒	Animals and other organisms
☒	☐	Human research participants
☒	☐	Clinical data
☒	☐	Dual use research of concern

Methods

n/a	Involved in the study	
☒	☐	ChIP-seq
☒	☐	Flow cytometry
☒	☐	MRI-based neuroimaging

Antibodies

Antibodies used	A table listing all antibodies is included in the manuscript, see Supplementary data file - Supplementary Table 1
Validation	Commercial antibodies were valided by selling company (see Supplementray Table 1). Laboratory made antibodies (Elp3 and Atat1) were valided on knockout mouse brain / knockdown fly heads / FD fibroblast samples. This is reported by western blotting for Elp3: in figure supplementary 1j, supplementary 2d, supplementary figure 4a of this paper; and for Atat1 in supplementary figure 1q of Even at aL,. Sci Adv (2019) Dec 18;5(12):eaax2705 .

Eukaryotic cell lines

Policy information about cell lines

Cell line source(s)	HEK293 cells, human primary fibroblasts. All relevant informations regarding these cell lines can be found in the Material and methods
Authentication	The HEK293 (Merck Chemicals) are routinely used in our lab and the fibroblast cell lines were purchased at Corriel (www.Corriell.org), they were authenticated and their description is reported in the Material and methods, section "human primary fibroblasts"
Mycoplasma contamination	Our cell lines were regularly tested and were negative for mycoplasma infection.

Commonly misidentified lines (See ICLAC register)	No commonly misidentified cell lines were used in the study

Animals and other organisms

Policy information about studies involving animals; ARRIVE guidelines recommended for reporting animal research

Laboratory animals	Mus musculus (C57BL/6J; Janvier Labs, Saint Berthevin, France) embryos (E14.5) and newborn of both sexes were used in the study; see methods of the manuscript for details, section "mice"; Drosophila melanogaster were used at 3rd instar larval stage or within the first week of life (both sexes).
Wild animals	No wild animals were used in the study.
Field-collected samples	No field collected samples were used in the study.
Ethics oversight	All experiments performed in this study adhere to all relevant ethical regulations for animal testing and research. The animal work was approved by the ethical committee of the University of Liege under the license #18-2056 and animals were treated according to the guidelines of the Belgian Ministry of Agriculture in agreement with the European Community Laboratory Animal Care and Use Regulations (86/609/CEE, Journal Official des Communautés Européennes L358, 18 December 1986).

Note that full information on the approval of the study protocol must also be provided in the manuscript.
